# Diagnostic Value of Sialic Acid in Pleural Effusion

**DOI:** 10.3779/j.issn.1009-3419.2010.04.15

**Published:** 2010-04-20

**Authors:** A BANSAL, S TANDON, S KHARB

**Affiliations:** Department of Biochemistry and Tuberculosis and Respiratory Medicine, Pt B D Sharma University of Health Sciences, Rohtak, India

**Keywords:** Lung neoplasms, ftoracic surgical procedures, Lymphatic metastasis, Treatment outcome

## Abstract

The present study was conducted in 30 patients of malignant pleural effusion and 30 patients of non malignant pleural effusion. Pleural fluid and blood samples were taken at the time of admission, before starting any treatment. Sialic acid levels were estimated in serum and pleural fluid by Warren's TBA method. In the present study, serum sialic acid levels were higher in group Ⅱ as compared to group Ⅰ. In the present study, pleural fluid sialic acid levels and PF/S ratio was higher in malignant pleural effusion (though difference was not statistically significant). Smokers in group Ⅱ had higher serum sialic acid as compared to group 1 (*P* < 0.05). fte sensitivity and specificity of pleural fluid/serum sialic acid ratio with cut off value of 0.7 were 76.67% and 20% respectively, while taking the cut off value of 70 mg/dL for pleural fluid sialic acid in malignant pleural effusions, the sensitivity was 63.33%, specificity 60% and positive predictive value 46.34%. ftese findings indicate that determination of sialic acid levels in pleural fluid has diagnostic value as a cheap, simple and reliable marker for malignant pleural effusion.

## Introduction

Malignant disease involving pleura is the second leading cause of exudative pleural effusions aTher parapneumonic effusions. It is one of the most common diagnostic problems requiring detailed investigation, encountered by specialist^[1]^. An increasing number of biochemical parameters have been reported to have diagnostic value in malignant pleural effusions ^[2-4]^. Sialic acid levels have been found to be elevated in neoplastic cells derived from lung, breast, stomach, and colon, and ovary, prostate and liver tumors^[5]^. Recently sialic acid has been reported to have a diagnostic value^[4]^. Hence, the present study is designed to measure sialic acid in pleural effusion and serum samples of patients in order to differentiate between malignant and nonmalignant diseases.

## Materials and methods

The present study was conducted in sixty patients attending Outpatient Department of Tuberculosis and Respiratory Medicine in collaboration with Department of Biochemistry, Pt BDS PGIMS, Rohtak, India. They were subdivided into two groups of thirty patients each: Group Ⅰ (patients with pleural effusion proved malignant by pleural biopsy) and Group Ⅱ (controls with nonmalignant pleural effusion). Inclusion criteria: sputum negative for AFB (at least on three occasions), confirmed malignant by pleural biopsy, exudative pleural effusion. All the cases were subjected to detailed clinical history, thorough clinical examination and routine investigations. Pleural fluid and blood samples were taken at the time of admission, before starting any treatment. Sialic acid levels were estimated in serum and pleural fluid by Warren's TBA method^[6]^. Data so obtained was analyzed statistically; student's *t*-test was applied and regression analysis was carried out.

## Observations

The clinical characteristics of the two groups are given in Tab 1. In group Ⅰ, there were 14 patients who were smokers, while in group Ⅱ there were 21 smokers. In group Ⅰ, 4 out of 30 patients were cases of benign pleural effusion, 18 had tubercular etiology, 10 had pneumonitis and 2 cases were with chylothorax. In group Ⅱ, 5 had malignant effusion, 25 had lung cancer, and 1 had mediastinal neoplasia.

**1:** Clinical characteristics

	Group Ⅰ (*n*=30)	Group Ⅱ (*n*=30)
Age	37.53	56.16
Sex	25M, 5F	19M, 11F
Smokers (%)	14 (46%)	21 (70%)
Pleural fluid protein (g%)	4.01±0.83	4.72±0.85
Group Ⅰ: patients with pleural effusion proved malignant by pleural biopsy; Group Ⅱ: patients with nonmalignant pleural effusion.		

Pleural effusion and serum sialic acid levels were higher in group Ⅱ as compared to group Ⅰ, though the difference was not statistically significant (Tab 2). PF/S ratio was higher in group Ⅱ as compared to group Ⅰ though difference was not statistically significant. Also, smokers had high pleural fluid and serum sialic acid levels and PF/S ratio as compared to nonsmokers. Smoker in groups Ⅱ had higher serum sialic acid as compared to group Ⅰ (*P* < 0.05).

**2:** Sialic acid levels in the two groups (Mean±SD, mg/dL)

	Group I				Group Ⅱ		
	Smokers	Nonsmokers	Total		Smokers	Nonsmokers	Total
Pleural fluid sialic acid	64.40±24.01	67.21±22.24	65.99±22.62		79.15±20.03	72.97±15.52	77.29±18.75
Serum sialic acid	73.76±21.87	80.04±18.43	77.14±20.03		81.73±21.95	90.20±13.48	84.75±20.12
Ratio	0.87±0.21	0.82±0.12	0.84±0.17		1.04±0.41	0.80±0.19	0.96±0.37

Taking cut off value of pleural fluid sialic acid as 65 mg/dL, in malignant pleural effusion, sensitivity was 63.33%, specificity 60% and positive predictive value 46.34% (Tab 3). Taking cut off value of 0.7 for PF/S sialic acid ratio, sensitivity was 76.67%, specificity 20%, and positive predictive value 48.94% in malignant pleural effusion.

**3:** Discriminative value of sialic acid level and PF/S ratio in both groups (%)

	Sensitivity	Specificity	Positive predictive value
Sialic acid >70 mg/dL	63.33	60.00	61.29
PF/S > 0.7	76.67	20.00	48.94
PF: Pleural fluid; S: serum.			

A positive correlation was observed between rise in sialic acid in pleural fluid and serum Sialic acid levels in group Ⅰ (Fig 1) and no significant correlation could be observed in group Ⅱ.

**1:**
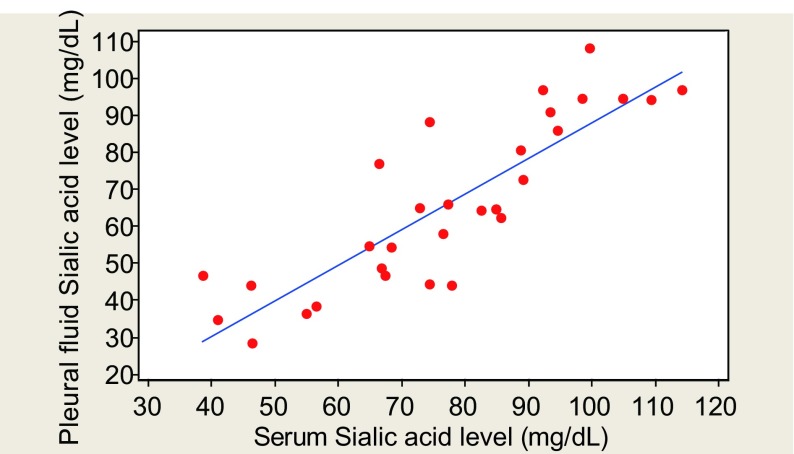
Correlation between PF and Serum Sialic acid in benign disease

## Discussion

In the present study, serum sialic acid levels were higher in group Ⅱ as compared to group Ⅰ. Changes in sialic acid content of patient's sera have been reported to reflect growth processes of benign and malignant character^[7]^. Raised serum sialic acid levels have been reported in various cancers^[5]^. Our findings are in agreement with those reported by Krolikowsky *et al*^[8]^Bektemur *et al*^[9]^ have reported high serum sialic acid levels in malignant pleural effusions though difference was not statistically significant. They also observed high pleural fluid sialic acid levels and PF/S ratio.

In the present study, pleural fluid sialic acid levels and PF/S ratio was higher in malignant pleural effusion (though difference was not statistically significant). Smokers in group Ⅱ had higher serum sialic acid as compared to group Ⅰ (*P* < 0.05).

These findings are in agreement with those reported in literature^[8-11]^. In the present study, the cases selected were of malignant pleural effusion in stage 4, so no correlation was done with stage of disease since our aim was to analyze sialic acid for differentiating malignant from nonmalignant pleural effusion.

In differential diagnosis of exudative pleural effusions, cytology is the most sensitive method. Since cytology findings are positive in half of such fluids, combined use of reliable tumor marker and cytology is a logical approach. Elevated concentration of sialic acid could be due to implants of malignant cells on pleura producing effusion as well as diffusion by pleural capillaries into pleural fluid.

On comparing sialic acid levels in serum and pleural fluid, 33.33% of cases of malignant pleural effusions had raised pleural fluid sialic acid (> 70 mg/dL) as compared to serum levels which were in the normal range. This could be either due to production and gradual absorption of sialic acid in diseased area or elevation of both serum and pleural fluid levels, but sialic acid disappears more slowly from pleural fluid.

Reports comparing sialic acid levels in serum in various histopathological types have found raised levels in squamous cell carcinoma and small cell carcinoma^[12]^, but, studies in pleural fluid are lacking. In the present study no specific correlation of sialic acid with any particular histopathological type could be observed.

Smokers had higher pleural fluid sialic acid levels in malignant pleural effusion as compared to non smokers (Tab 2, *P* > 0.05). While in benign pleural effusion pleural fluid sialic acid levels were higher in non smokers (Tab 2, *P* > 0.05). PF/S ratio was higher in smokers than in non smokers in malignant pleural effusion and lower in smokers in benign pleural effusions (Tab 2, *P* < 0.05, *P* > 0.05, respectively).

Serum sialic acid levels have been reported to be increased in smokers and alcoholics since smoking induces tissue inflammation and is a known carcinogen^[13]^. Hence elevated sialic acid levels in pleural fluid could be attributed to smoking and this could possibly a secondary process to malignancy that needs to be established by further studies. Since the studies have reported that total sialic acid levels remain unchanged following one year of smoking cessation^[13, 14]^.

In the present study, a significant positive correlation was observed between pleural fluid and serum sialic serum sialic acid levels in benign pleural effusion (Fig 1). A positive correlation was also observed between pleural fluid and serum sialic acid levels in malignant pleural effusion though difference was not statistically significant. Bektemur et al had also observed no significant correlation between pleural fluid to serum lipid bound sialic acid ratios in benign and malignant groups^[9]^.

Taking cut off value of 70 for pleural fluid sialic acid in malignant pleural effusions, sensitivity was 63.33%, specificity 60% and positive predictive value 46.34% (Tab 3).

Taking cut off values of > 0.7 for PF/S ratio, sensitivity was 76.67%, specificity 20% and positive predictive value 48.94% (Tab 2). These findings are in agreement with those reported in literature^[10]^. Thus, pleural fluid sialic acid in malignant pleural effusion along with PF/S ratio have good sensitivity and positive predictive value and can prove to be a reliable marker in differentiating benign and malignant pleural effusion.
